# Leveraging pre-trained AI models for robust promoter sequence design in synthetic biology

**DOI:** 10.52601/bpr.2025.240033

**Published:** 2026-04-30

**Authors:** Gui Yang, Yijie Chen, Qinghua Guo, Xinyang Li, Zhen Zhou

**Affiliations:** 1 Nanjing Drum Tower Hospital Center of Molecular Diagnostic and Therapy, State Key Laboratory of Pharmaceutical Biotechnology, Jiangsu Engineering Research Center for MicroRNA Biology and Biotechnology, NJU Advanced Institute of Life Sciences (NAILS), School of Life Sciences, Nanjing University, Nanjing 210023, China; 2 Institute of Artificial Intelligence Biomedicine, Nanjing University, Nanjing 210023, China

**Keywords:** Promoters, AI, Pre-train models, Synthetic biology

## Abstract

Although artificial intelligence (AI) has begun to be applied in synthetic biology, it is limited by its reliance on large amounts of high-quality data, which presents a significant challenge in synthetic biology. Pre-trained models have profoundly influenced natural language processing by enabling systems to understand and generate human language with remarkable accuracy and efficiency by capturing complex linguistic patterns and contextual nuances. This study applies the concept of pre-trained models to promoter sequence analysis through an innovative pre-training and fine-tuning paradigm. Our analysis reveals that pre-trained DNA models, particularly DNABERT, consistently outperform non-pre-trained models in predicting promoter expression levels across various dataset sizes. Building on DNABERT's strengths, we developed the AI model Pymaker, which specializes in predicting yeast promoter expression levels. Additionally, we introduced a novel base mutation model to simulate promoter mutations, enabling the generation of new promoter sequences. By integrating Pymaker with this mutation model, we effectively screened for high-expression, mutation-resistant promoters. Experimental validation in Saccharomyces cerevisiae showed that these selected promoters significantly enhanced LTB protein expression. Notably, Pymaker’s predictions demonstrated superior accuracy, achieving a three-fold increase in protein expression compared to traditional promoters. Our findings highlight the potential of Pymaker not only to identify robust promoters but also to significantly reduce reliance on conventional, labor-intensive experimental methods, heralding a new era in synthetic biology and genetic engineering with practical applications in biopharmaceuticals.

## INTRODUCTION

Although artificial intelligence (AI) has begun to be applied in synthetic biology, it is limited by its reliance on large amounts of high-quality data, which presents a significant challenge in synthetic biology. Consequently, deep learning models face issues such as insufficient training data, data imbalance, uncertainty propagation, catastrophic forgetting, overfitting, and vanishing gradients (Alzubaidi *et al.* 2021; Caviglione *et al.* 2023; Goshisht 2024; Johnson and Khoshgoftaar 2019).

In synthetic biology, designing functional promoters is an important and challenging task. The strength of core promoters is influenced by various factors, including component type and arrangement, transcription factor binding sites, and upstream and downstream regulatory elements (Browning *et al.* 2019; Butler and Kadonaga 2002; Lenhard *et al.* 2012; Maston *et al.* 2006; Vo Ngoc *et al.* 2017). Traditional promoter design methods primarily rely on site-directed mutagenesis and random mutagenesis (Levine and Tjian 2003). However, these methods are not only time-consuming and costly but also offer limited predictability regarding which mutations will yield the desired functions (Browning and Busby 2004; Kosuri and Church 2014). By leveraging gene synthesis and DNA assembly technologies, together with computational modeling and machine learning algorithms, synthetic biology can expedite the optimization process of promoters (Curran and Alper 2012; Redden and Alper 2015). Various AI models have already been utilized for promoter prediction (de Boer *et al.* 2020; Vaishnav *et al.* 2022). Despite their effectiveness, these models still require large datasets for training. Therefore, the pressing issue remains: how to precisely design promoters using limited biological sample data.

The pre-training and fine-tuning paradigm has significant applications in the creation of AI models. Initially, pre-training is conducted on large-scale, unlabeled, or minimally labeled datasets, allowing the model to learn general feature representations. This is followed by fine-tuning on specific task datasets to meet particular needs. In the field of life science research, foundational models such as BioBert and DNABERT have already emerged, demonstrating considerable potential and effectiveness in various applications (Ji *et al.* 2021; Lee *et al.* 2020). Because broad data patterns are captured during the pre-training stage, it is possible to achieve high performance even when applied to fine-tuning tasks with smaller data volumes.

In this study, we employed the pre-training and fine-tuning paradigm to compare the performance of various biological pre-trained models in predicting promoter expression levels under different sample sizes. We developed an AI model termed Pymaker to predict promoter expression levels. Additionally, considering the potential for base mutations in promoters during yeast cultivation, we developed a base mutation model to simulate base mutations occurring in the cell culture process and generate new promoter sequences. By combining Pymaker with the base mutation model, we were able to rapidly screen for high-expression promoters that are resistant to mutations—a feat difficult to achieve under natural conditions.

## RESULTS

### Evaluation of different pre-trained models’ prediction performance with varying training sample sizes

**Figure 1:**
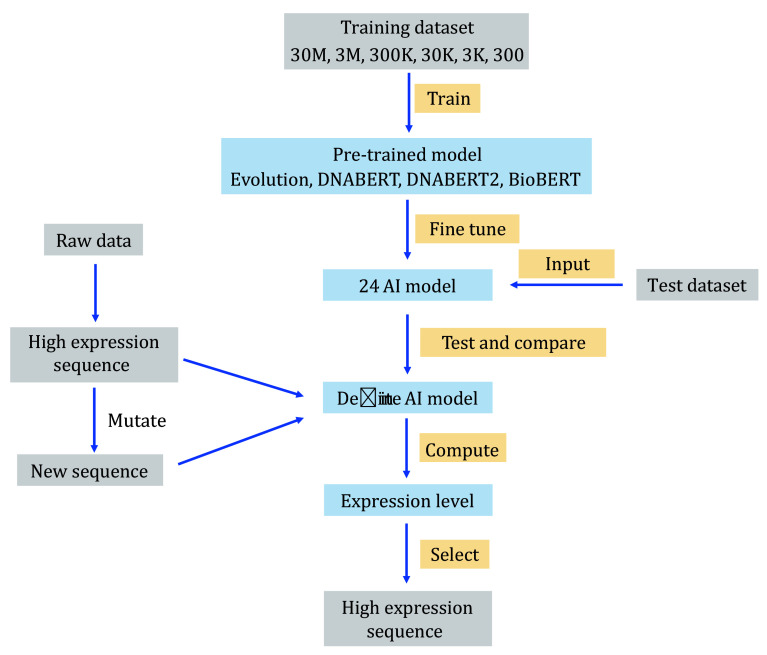
Schematic diagram of screening high-expression mutation-resistant promoters based on pre-training model

To compare the performance of different pre-trained models, we designed an experimental workflow. As shown in Fig. 1, we used the dataset from Vaishnav *et al*., consisting of 30 million samples, as the initial training dataset. Additionally, we utilized a high-precision experimental dataset with 61,150 samples as the test set. To compare the models’ performance with varying training sample sizes, we randomly selected datasets of 300, 3000, 30,000, 300,000, and 3,000,000 samples from the 30 million dataset for training. For pre-trained models, we selected three models: DNABERT, DNABERT2, and BioBERT. These base models were fine-tuned using different training sample sets. The models’ performance was evaluated using the Pearson Correlation Coefficient (PCC), which measures the correlation between the predicted promoter sequence expression levels and the actual experimental measurements. We then compared these results with the Evolution model from the Vaishnav *et al*. paper. Sequences with expression levels represented as integers were removed from the original dataset because they were detected in only a single culture dish and introduced significant errors. Subsequently, the training was conducted on datasets of varying sizes, specifically 300, 3000, 30,000, 300,000, 3,000,000, and 30,000,000 sequences. These sequences were randomly selected from the total dataset of 30,000,000 sequences after the removal of integer data points.

**Figure 2:**
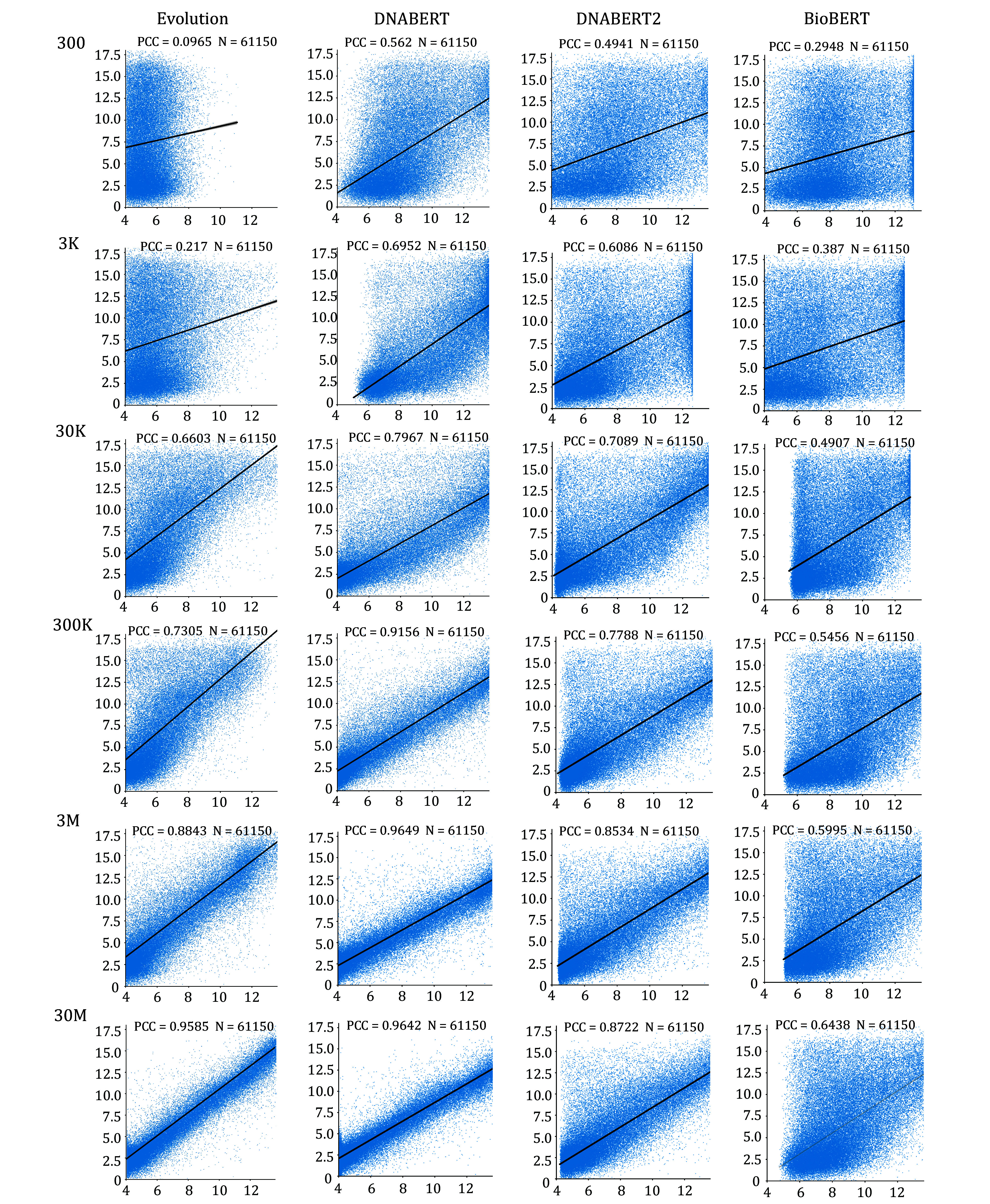
Performance of AI models trained with different pre-trained models and varying sample sizes. The figure presents four distinct AI models: Evolution, DNABERT, DNABERT2 and BioBERT. The left column lists the sample sizes of different training datasets. Except for the 30M sample, the other samples were randomly selected from the training datasets with non-integer expression levels removed. The scatter plots in the center display the prediction results of each AI model on the same test dataset (*N* = 61,150). Each scatter plot column represents the AI model used, and each row indicates the sample size of the training dataset. Since the test dataset is identical across plots, the number of data points is consistent in each scatter plot. The *x*-axis represents the predicted expression levels, while the *y*-axis shows the actual measured values

Results indicate that the four models achieved the highest fit when trained on the dataset with 30M samples (Fig. 2). Their respective PCCs were 0.9585, 0.9642, 0.8722, and 0.6438. Notably, DNABERT achieved a PCC of 0.9642, slightly surpassing the Evolution model's 0.9585 (with the Vaishnav *et al*. paper reporting a PCC of 0.963). This demonstrates that we successfully replicated the results from the original study and that our AI models, constructed using the pre-training and fine-tuning paradigm, outperformed the Evolution model that was not pre-trained.

As the sample size decreases, the fit of all four models gradually deteriorates. For the datasets with 3M and 300K samples, the PCCs achieved by the DNABERT model were 0.9469 and 0.9156, while the Evolution model attained PCCs of 0.8843 and 0.7305. Our research findings indicate that when using a smaller sample size of 3M and 300K, the DNABERT pre-trained model exhibits a higher degree of fit compared to the non-pre-trained Evolution model.

As the training sample size drastically drops to 3000, we compare the fitting accuracy of the four models. The PCC for Evolution, DNABERT, DNABERT2, and BioBERT were 0.217, 0.6952, 0.6086, and 0.387, respectively. Models trained with the Evolution and BioBERT pre-trained models resulted in highly dispersed data points, with substantial discrepancies between the measured and predicted expression levels for nearly all data points. The fit of these models was poor, with PCCs below 0.5, rendering them unusable. Conversely, the DNABERT pre-trained model achieved the highest PCC of 0.6952. When the sample size is reduced to 300, only the DNABERT model had a fitting accuracy greater than 0.5, whereas the other three models fell below this threshold. Notably, the Evolution model had a particularly poor fit with a PCC of just 0.0965. This indicates that in extremely small sample scenarios, the DNABERT pre-trained model still holds some predictive value, whereas the non-pre-trained Evolution model proves entirely unusable.

In addition, we also calculated the Mean Squared Error (MSE) and the Coefficient of Determination (*R*²) for the regression models, as detailed in Table 1. As shown in Table 1, the AI models developed based on the DNABERT pre-trained model performed optimally in terms of MSE and R² for sample sizes of 300, 30,000, 300,000, and 3 million. This indicates that the AI models utilizing DNABERT as the pre-trained model consistently achieved the best performance.

**Table 1:** The fitness of 24 pre-trained models was evaluated using different statistical metrics

	Evolution				DNABERT				DNABERT2				BioBERT		
	MSE	*R* ^2^	PCC		MSE	*R* ^2^	PCC		MSE	*R* ^2^	PCC		MSE	*R* ^2^	PCC
300	22.165	−0.1497	0.0965		**16.1507**	**0.1622**	**0.562**		16.2006	0.1596	0.4941		22.5675	−0.1706	0.2948
3K	19.8269	−0.0284	0.217		19.5606	−0.0146	**0.6952**		**13.4906**	**0.3002**	0.6086		17.6785	0.0829	0.387
30K	11.6729	0.3944	0.6603		**10.4669**	**0.457**	**0.7967**		10.6473	0.4476	0.7089		17.2544	0.1049	0.4907
300K	10.2928	0.466	0.7305		**4.9601**	**0.7427**	**0.9156**		9.1765	0.5239	0.7788		18.9475	0.0171	0.5456
3M	5.1385	0.7334	0.8843		**4.6759**	**0.7574**	**0.9469**		7.64	0.6036	0.8534		15.7163	0.1847	0.5995
30M	**3.6328**	**0.8115**	0.9585		4.1865	0.7828	**0.9642**		7.4754	0.6122	0.8722		15.9604	0.1721	0.6438
Note: The bold numbers in the table represent the State-of-the-Art scores for all models under the evaluation metrics (MSE, R2, PCC) when the sample size of the training data is fixed															

It can be observed that AI models constructed using the pre-training and fine-tuning paradigm have significant advantages with small sample sizes. The predictions from the AI model based on DNABERT closely align with the actual results, whereas the Evolution model becomes entirely unusable with small sample sizes. This indicates that our pre-training and fine-tuning paradigm can effectively address the issue of insufficient sample sizes. Therefore, we utilized DNABERT as the pre-trained model and trained it with a dataset comprising 30 million samples to construct an AI model capable of predicting yeast promoter expression levels—termed Pymaker for subsequent research.

### Screening for high-expression, mutation-resistant promoter sequences based on the Pymaker model

Due to the potential for random mutations in promoters after multiple generations of cultivation, expression levels may change (Maston *et al.* 2006; Milito *et al.* 2023). Traditionally, the optimal promoters are screened experimentally, but this approach is time-consuming, labor-intensive, and costly (Browning and Busby 2004; Kosuri and Church 2014). To address this issue, we designed a base mutation model to simulate the promoter mutation process. We input the generated mutated promoter sequences into Pymaker to predict their expression levels and plotted the expression intensity variation curves for each generation of mutated promoters. This approach demonstrates the changes in expression intensity under multiple generations of random mutations.

Since many artificially synthesized core promoter sequences lack standard structures, existing mutation hotspots and preferences cannot be applied to our mutation model. Therefore, we assumed that mutations occur purely at random. We referenced studies that also adopted the random mutation hypothesis to construct our base mutation model. We subjected the top 1000 sequences ranked by expression levels to 100 generations of random mutations.

**Figure 3:**
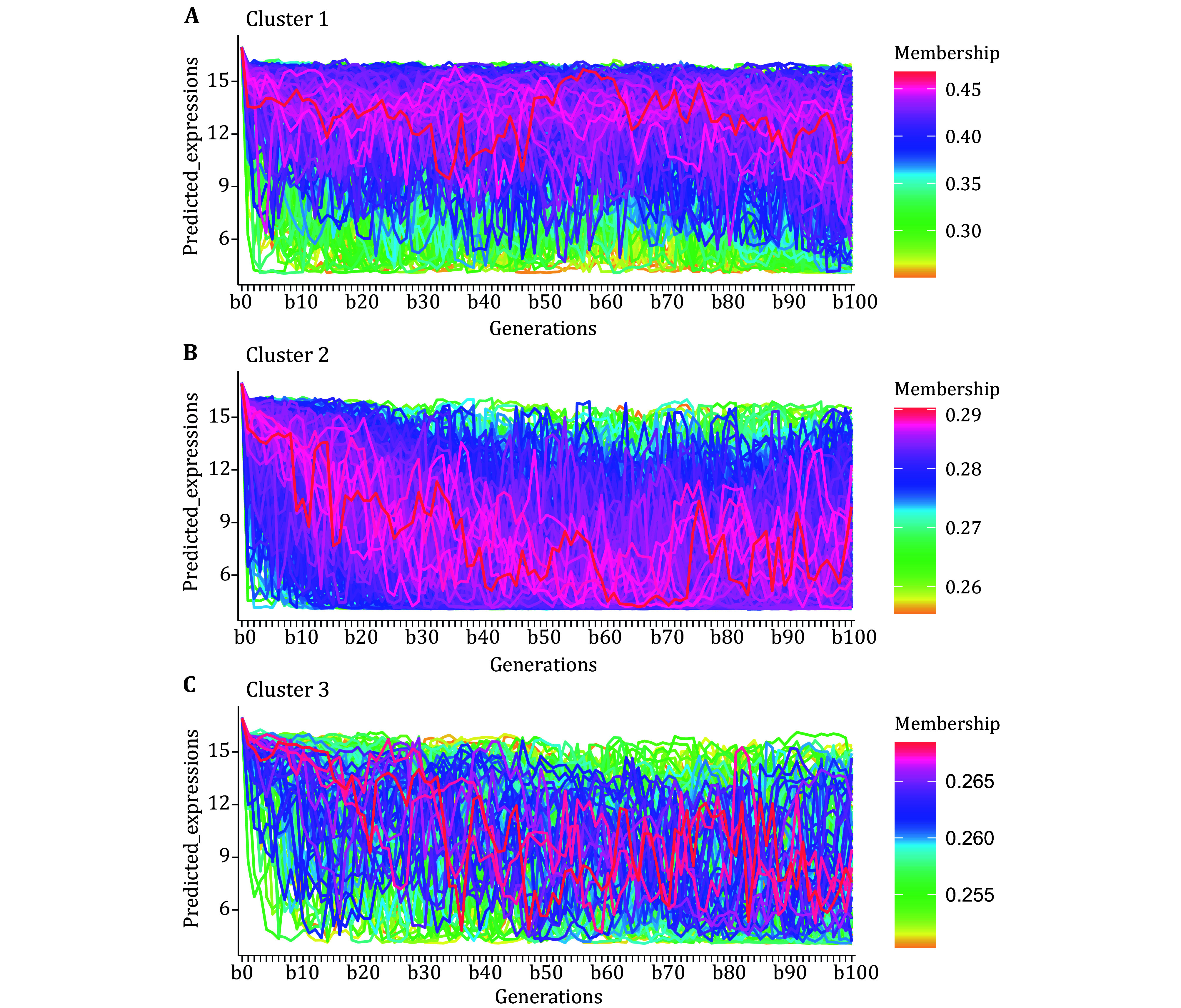
The changes in expression levels of 1000 high-expression promoters after 100 generations of mutation. **A** Cluster 1 consists of high-expression promoters that maintain elevated expression levels. **B** Cluster 2 encompasses high-expression promoters that experience a significant reduction in expression levels following mutations, ultimately settling at low expression levels. **C** Cluster 3 includes high-expression promoters that exhibit unstable expression levels after mutations. The *y*-axis represents the predicted expression levels, and the *x*-axis indicates the number of generations, ranging from 0 to 100. Darker lines correspond to denser curves. The red region indicates the highest line density, while the yellow region indicates the lowest line density. Each line represents a single promoter

Based on this mutation model, we obtained the mutated sequences for each generation and input them into the AI model to predict the expression level changes of the top 1000 high-expression promoters under multiple generations of random mutations. As shown in Fig. 3, the 1000 high-expression promoter sequences can be divided into three clusters. In Fig. 3, darker lines represent denser curves, with the highest line density in the red region and the lowest in the yellow region. We can observe that during 100 generations of random mutations, the red curves in Cluster 1 are concentrated at the top, indicating that most promoters in Cluster 1 maintain high expression levels. In Cluster 2, the red curves are concentrated at the bottom, suggesting that most promoters in Cluster 2 experience a significant drop in expression levels after mutation, eventually reaching low expression levels. In Cluster 3, the red curves fluctuate, indicating that most promoters in Cluster 3 have unstable expression levels post-mutation, showing significant increases and decreases. The high-expression and mutation-resistant promoters in Cluster 1 are what we need.

### Screening of high-expression promoter sequences for LTB protein production

*Escherichia coli* heat-labile enterotoxin B (LTB) is an important oral vaccine adjuvant and intestinal adhesion enhancer. Conjugating LTB with antigens can enhance the immune response of vaccines. However, the expression level of LTB has consistently been low, which limits its widespread application. We aim to use Pymaker to screen for mutation-resistant promoter sequences that can enhance LTB expression levels.

**Figure 4:**
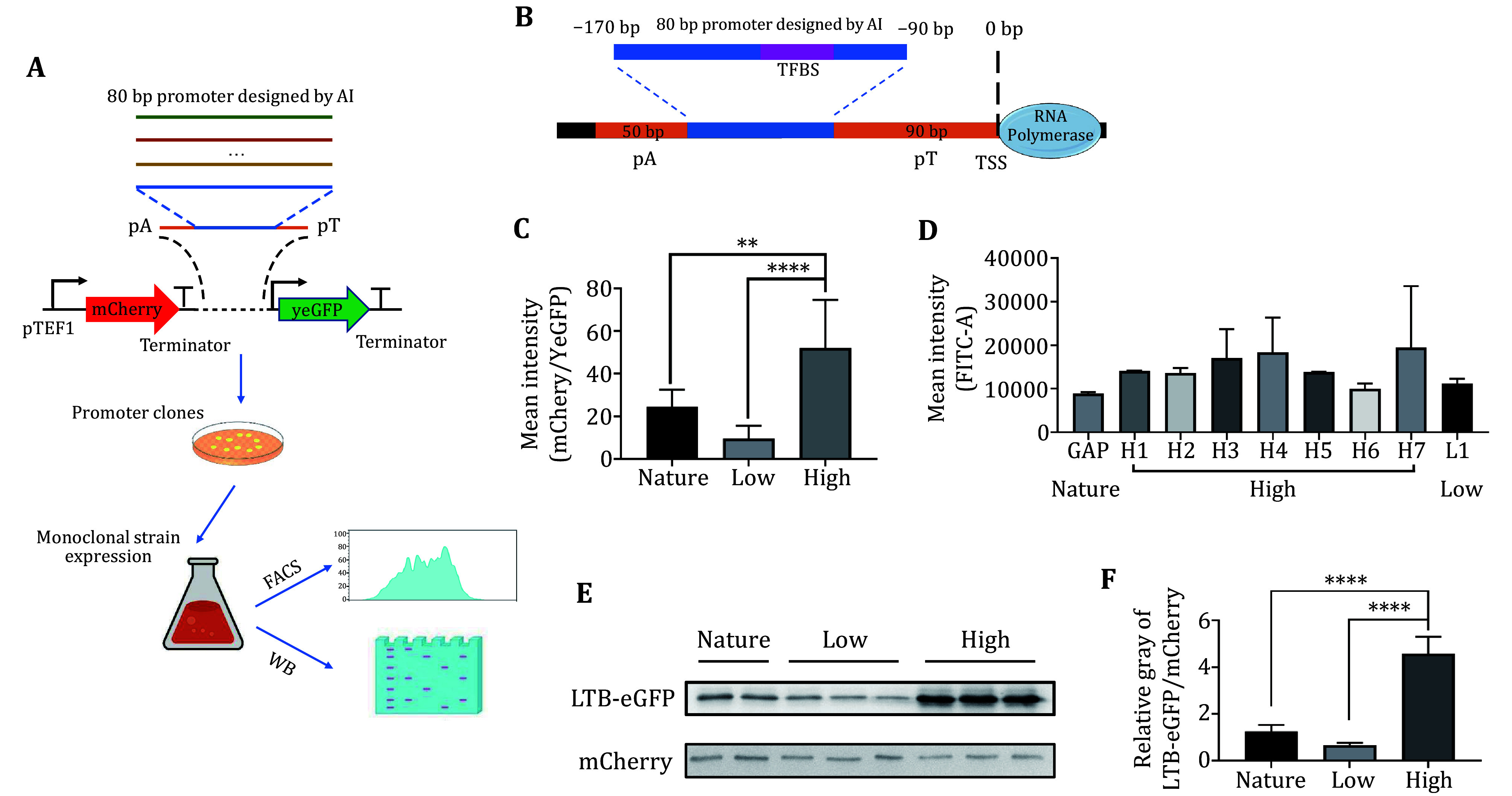
Screening high-expression promoters generated by Pymaker for expressing LTB protein. **A** Schematic of the experimental workflow. **B** Schematic Diagram of Promoter Design. TSS: suspected Transcription Start Site, TFBS: Transcription Factor Binding Site **C** Expression levels of different types of promoters detected using a dual fluorescence reporter system. Mean intensity is calculated by the geometric mean of each population’s fluorescence intensity examined by flow cytometry. Unpaired *t*-test, **p < 0.01, ****p < 0.0001. **D** FACS analysis to measure LTB-eGFP expression levels via fluorescence intensity. **E** Western blot analysis of LTB-eGFP expression driven by different types of promoters. **F** Quantification of LTB-eGFP expression from Western blot results through grayscale value analysis. Unpaired *t*-test, ****p < 0.0001

Initially, we experimentally demonstrated that random DNA yields diverse expression levels in a yeast promoter library. To robustly quantify promoter activity, we used an episomal dual reporter system expressing a constitutive yeast-enhanced green fluorescent protein (yeGFP) and a variable mCherry protein. The system can eliminate the effects of plasmid copy number and yeast host growth conditions, allowing for the direct measurement of the relative expression intensity of the promoters (Kinney *et al.* 2010; Kosuri *et al.* 2013; Shalem *et al.* 2015). We created ten synthetic promoter scaffolds and one based on the native ADH1 promoter sequence, each consisting of 50–90 bp of constant scaffold sequence flanking 80bp of random DNA (−170 to −90, relative to the presumed transcription start site (TSS) (Fig. 4B). We predicted and generated 80bp high-expression, mutation-resistant promoter sequences and selected seven of these high-expression promoters for subsequent experimental validation. Referencing the study by Vaishnav *et al*. (Vaishnav *et al.* 2022), the 80 bp promoter sequences were inserted into the ADH1 promoter framework poly-T(pT) and poly-A(pA) sequences and linked to a dual fluorescence reporter system (Fig. 4A).

The performance of the candidate promoters was first evaluated in expressing the yeGFP gene. We constructed plasmids capable of expressing both the yeGFP gene and the mCherry gene, where the yeGFP gene was driven by the predicted promoters, and the mCherry gene was driven by the TEF1 promoter. Nine plasmids (one with a natural promoter, seven with high-expression promoters, and one with a low-expression promoter) were individually transformed into *Saccharomyces cerevisiae*. Using FACS technology, we measured the expression levels of the promoters by the GFP/mCherry fluorescence ratio. The results showed that the predicted high-expression promoters significantly outperformed the natural promoter, validating the predictions made by Pymaker (Fig. 4C).

Next, we constructed a plasmid expressing a conjugated LTB-eGFP protein to assess the performance of our screened promoters in expressing the LTB protein. We created nine pDual-LTB-eGFP plasmids, each containing either high-expression promoters (H1-H7), a low-expression promoter (L1), or a natural promoter (GAP). FACS analysis indicated that the expression level of the high-expression promoter H7 was the highest (Fig. 4D). Using Western blot for quantitative detection, we compared the protein expression levels driven by the high-expression promoter H7 and the low-expression promoter L1 (Fig. 4E). The results demonstrated that the LTB-eGFP fusion protein was successfully expressed in yeast, with the expression level driven by the high-expression promoter being significantly higher than that of the natural promoter, by a factor of three (Fig. 4F). These findings confirm the effectiveness of Pymaker in predicting high-expression promoters.

## DISCUSSION

In this study, we explored the application of artificial intelligence in promoter sequence research based on a paradigm of pre-training and fine-tuning. By comparing the performance of DNABERT, DNABERT2, and BioBERT pre-trained models with different scales of training datasets, we found that pre-trained models consistently outperformed non-pre-trained models in terms of fitting accuracy. Particularly, DNABERT and DNABERT2 showed significantly higher performance on small training datasets compared to other models.

Notably, we intentionally used BioBERT as a control to demonstrate that not all pre-trained models can effectively address the issue of data insufficiency in life science research. Our results show that BioBERT's performance is indeed significantly lower than that of other models, likely due to the fact that it was pre-trained on biomedical corpora (natural language) with limited DNA sequence information. This finding underscores the importance of selecting pre-trained models that are relevant to the specific task at hand and have been trained on sufficient amounts of similar data.

Moreover, our study highlights the potential advantages of using pre-trained models in small-sample or zero-shot tasks, but also emphasizes the importance of exposure to large amounts of similar data during pre-training. We found that even if a pre-trained model has an advantage in small-sample or zero-shot tasks, its performance can be compromised if it lacks sufficient knowledge about the specific domain or type of data being modeled.

Our research also revealed that despite DNABERT2 being an improved model over DNABERT, DNABERT still outperformed DNABERT2 in predicting promoter sequence expression capabilities. We speculate that although DNABERT2 introduced some improvements in the tokenization process of DNA sequences and incorporated DNA sequences from more species, the k-mer tokenization method used in DNABERT may be more suitable for promoter sequence tasks.

There remains considerable potential for improving datasets to enhance the application of pretrained models in synthetic biology. While our research has been constrained to *Saccharomyces cerevisiae*, there are concerns about how shifting the biological context might affect the results. Nonetheless, our paradigm of pre-training and fine-tuning offers a robust and efficient framework for experimentation across various organisms, provided that experimental data at a scale of thousands is available. Highly robust AI models can, to some extent, help us replace complex and expensive experiments. By simulating biological processes using AI models, we can quickly accumulate a large amount of biological simulation data, allowing us to uncover important biological patterns and principles.

Using a mutation model, we identified three major types of promoter sequences in terms of their mutation resistance. One type can effectively resist mutations in the sequence. These promoters could potentially be used for the expression of important housekeeping genes. The other two types of promoter sequences cannot effectively resist mutations. A small portion of these promoters show fluctuating expression levels during mutations, which could suggest their role in expressing luxury genes, such as resistance genes. The presence of such promoter sequences likely contributes to the species' ability to adapt to different environments. Our mutation model has the potential to elucidate the sequence features associated with various anti-mutation properties. We plan to validate these findings by assessing mutation resistance levels *in vivo* in future studies.

In the fields of biopharmaceuticals and genetic engineering, the stability and mutation resistance of promoters are equally crucial. The promoters designed by Pymaker demonstrated excellent mutation resistance in the yeast expression system, indicating that these promoters possess higher stability and reliability in practical applications. In future research, we can incorporate more experimental data on promoter expression strength from other species, enabling Pymaker to be utilized across various applications in synthetic biology.

The AI-based promoter design technology developed in this study, while demonstrating significant potential in synthetic biology applications, has also raised several ethical concerns. Primarily, the generation and optimization of promoter sequences using AI may result in unintended biological functions, which could disrupt ecological systems. Additionally, although the predictive capabilities of AI models are robust, they are inherently dependent on specific training datasets, which could lead to biases and unforeseen consequences. Therefore, it is critical that these AI tools are used responsibly during their development and application (Kurtoğlu *et al.* 2024). To address these ethical challenges, it is recommended that researchers adopt transparent and rigorous model development processes, ensuring that the generated sequences are thoroughly validated under various conditions to minimize the risk of unintended functions. Moreover, the application of such technologies should strictly adhere to existing biosafety and bioethics guidelines, avoiding widespread use without comprehensive risk assessments.

## METHODS

### Datasets

The original training data were entirely sourced from the study published by Vaishnav *et al*. (Vaishnav *et al.* 2022), with all measurements taken in complex media. The test dataset used was also the same one employed by Vaishnav *et al*. in the same study (Vaishnav *et al.* 2022). The available training dataset can be accessed at: https://codeocean.com/capsule/8020974/tree/v1/data/Glu/training_data_Glu.txt.

The available test dataset can be accessed at: https://codeocean.com/capsule/8020974/tree/v1/data/test_data/All_promoter_fragments_Native.csv.

### Model training based on pre-trained DNA models

For the pre-trained DNABERT model (Ji *et al.* 2021), a random selection of 3,000,000 data points from the entire raw training dataset was used for the pre-experiment. A common learning rate (1 × 10^−5^) was used to ensure the functionality of the pipeline. Based on the learning rate, various k-mer values (3, 4, 5, 6) were tested, and it was determined that a k-mer of 4 yielded the best results. Based on the k-mer of 4, various optimizers (SGD, Adam, Adamax, RMSprop, Adagrad) were tested, and Adam was selected as the optimizer, according to its best performance. To address overfitting, weight decay was implemented as a regularization technique. Moreover, we fine-tuned the models with a combination of different learning rates (1 × 10^−3^ to 1 × 10^−7^), weight decay (0.005–0.1) and batch size, specifically for each data group. The AI model based on the DNABERT pre-trained model was implemented using Python 3.8, Transformers version 4.28.0, and Pytorch version 1.8.0.

For DNABERT2 and BioBERT, fine-tuning was conducted using the same method. The AI model based on the DNABERT2 pre-trained model was implemented using Python 3.8, Transformers version 4.30.2, and Pytorch version 1.13.1. The AI model based on the BioBERT pre-trained model was implemented using Python 3.8, Transformers version 4.24.0, and Pytorch version 1.13.0. Leveraging 4 NVIDIA RTX A6000 GPUs, these BERT models were fine-tuned by using the full training dataset (30 million), taking a total of about nine days to complete ten epochs.

The Evolution model was constructed and trained using the architecture outlined in the previous paper (Vaishnav *et al*. 2022). To leverage GPU acceleration for the training process, a transition from TensorFlow version 1 to version 2 was performed. In the script, only minor code modifications were made to ensure compatibility with the updated environment, while other components, such as the neural network architecture, remained unchanged. Ultimately, Evolution was implemented using Python 3.7, Tensorflow-estimator version 2.4.0, and Tensorflow-gpu version 2.4.0. Leveraging 1 Tesla T4 GPU, the Evolution model was fine-tuned by using the full training dataset (30 million), taking a total of about six days to complete ten epochs.

### Base mutation model

In natural environments, core promoter sequences are altered by point mutations and indels (insertions and deletions), which subsequently impact their expression levels. Given that our model’s input is a short core promoter sequence, we focus here exclusively on the effects of point mutations on the expression strength of this core promoter. We constructed a base mutation model aiming to accelerate the evolutionary process by introducing numerous point mutations, allowing us to investigate whether certain clusters emerge within these core promoter sequences under mutation pressure.

The top 1000 core promoter sequences with the highest expression levels, identified by the best-performing AI model, were subjected to 100 generations of random mutations. Here, “generation” does not refer to traditional culture generations but to mutation iterations (an event where one or several bases undergo a mutation due to internal or external factors). We introduced 1–3 random mutations at random sites per generation in core promoters. The mutated sequences from each generation were then input into the best-performing AI model to predict their expression levels.

### Statistics and analysis

The performance of the AI model was evaluated using the Pearson correlation coefficient (PCC), mean squared error (MSE), and *R*². Higher PCC and *R*² values, closer to 1, indicated a higher correlation between the variables and better model training effectiveness, while lower MSE values indicated better model performance.

### The construction of plasmid

The dual-reporter vector pDual-mCherry-yeGFP sequence was obtained from the article published by Vaishnav *et al*. in *Nature* (AddGene Plasmid #127546). Plasmids containing different promoters (H1-H7 and L1 sequences in supplementary Table S1) were synthesized by a company. Based on the pDual-mCherry-yeGFP plasmid, linearized vectors were formed through Xho I and BamH I digestion from NEB, followed by the insertion of the PCR-amplified LTB sequence (primers listed in supplementary Table S1). These constructs were transformed into *E. coli* DH5α, plated, and cultured at 37°C for 12 h. A single colony was picked, expanded in liquid culture, and the plasmids were extracted and subjected to Sanger sequencing to obtain the complete pDual-LTB-eGFP plasmid.

### Fluorescence activated cell sorting (FACS)

Yeast cells successfully expressing the dual fluorescent reporter protein and LTB-eGFP fusion protein were collected and washed with PBS, adjusting the cell suspension concentration to 1–5 × 10^6^ cells/mL. Fluorescence-activated cell sorting (FACS) analysis was performed using a flow cytometer equipped with 488 nm and 588 nm excitation lasers. Data were analyzed using FlowJoV10 software to determine fluorescence intensity.

### Western blot

Protein extraction was carried out following the instructions provided in the Yeast Total Protein Extraction Kit (SK2440) from COOLABER SCIENCE. SDS-PAGE gels were prepared using the Color PAGE Gel Rapid Preparation Kit (28X01) from Yeasen Bio. Protein samples were separated on 12% SDS-PAGE gels and transferred onto nitrocellulose membranes (Hybond™ from Cytiva Life Sciences). The membranes were blocked with 5% non-fat milk for 2 h. The target proteins were incubated overnight at 4°C with the primary antibody (rabbit anti-LTB). After washing three times with Washing Buffer on a shaker for 10 min each, the membranes were incubated with HRP-conjugated secondary antibody at room temperature for 90 min. Following three additional washes with Washing Buffer on a shaker for 10 min each, the target proteins were developed using a chemiluminescent substrate in a darkroom. The band intensities of the target proteins were measured using Image J software, and the grayscale values were calculated.
